# Extensive intra‐myocardial calcifications: Value of multimodality imaging

**DOI:** 10.1111/echo.15357

**Published:** 2022-05-03

**Authors:** Fabiola B. Sozzi, Laura Iacuzio, Marco Schiavone, Filippo Civaia, Stefano Carugo, Ciro Canetta, Franck Levy, Armand Eker

**Affiliations:** ^1^ Fondazione IRCCS Ca' Granda Ospedale Maggiore Policlinico Milan Italy; ^2^ Cardiothoracic Centre CCM Monaco MC; ^3^ Cardiology Unit Luigi Sacco University Hospital Milan IT

**Keywords:** cardiac CT, cardiac MRI, intra‐myocardial calcifications

## Abstract

**Background:**

Massive myocardial calcification is a very rare finding.

**Introduction:**

Accurate identification and characteriation may help the clinicians to determine the etiology and clinical significance.

**Results:**

In this case, the diagnostic pathway excluded previous myocardial infarction, myocarditis, and calcium‐phosphate disorders. A possible dystrophic etiology was considered.

**Discussion:**

There are no standardized imaging features available to classify specific subtypes of intra‐myocardial calcifications. The relative merits of computed tomography and cardiac magnetic resonance (CMR) in providing complimentary diagnostic information in the evaluation of calcific myocardial lesions are shown.

**Conclusion:**

Knowledge of the potential etiology and their imging patterns are important to provide a concise and accurate differential diagnosis.

## 1 INTRODUCTION

**FIGURE 1:**
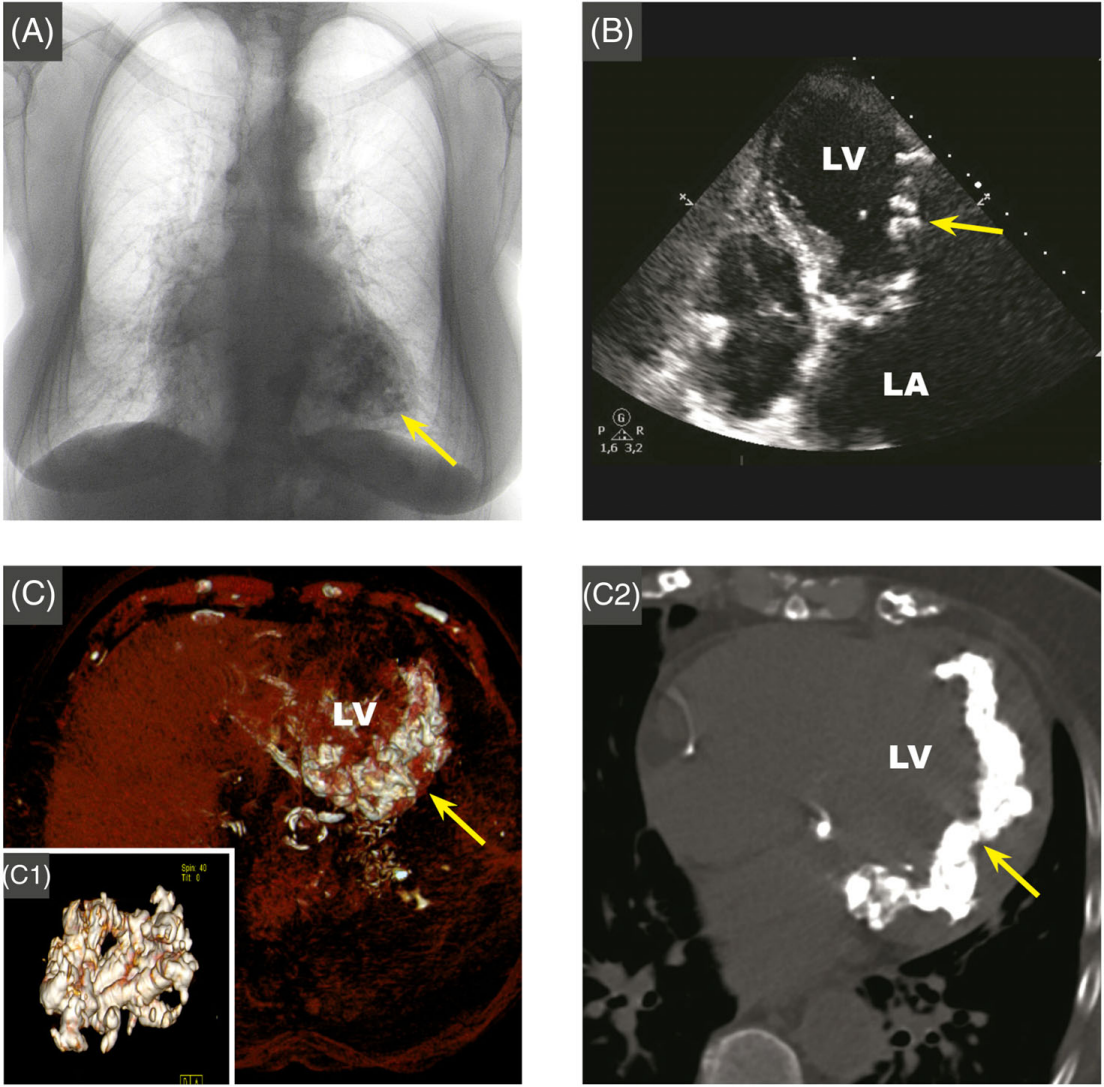
(A) Chest X‐ray showing a diffuse hypodense lobulated area in the left ventricle. (B) Video 1. 2D‐Echocardiogram revealing diffuse aortic and mitral calcifications with mild stenosis, marked septum and antero‐lateral asymmetric hypertrophy, with extensive antero‐lateral calcifications. (C) C1: CT‐VRT reconstruction of the left ventricle, respectively axial view and focus on the coronal view. C2: Video 2. Non‐contrast CT MPR‐axial view presents widespread calcifications in the left ventricular wall extended to the mitral‐aortic annulus and both coronary arteries

**FIGURE 2:**
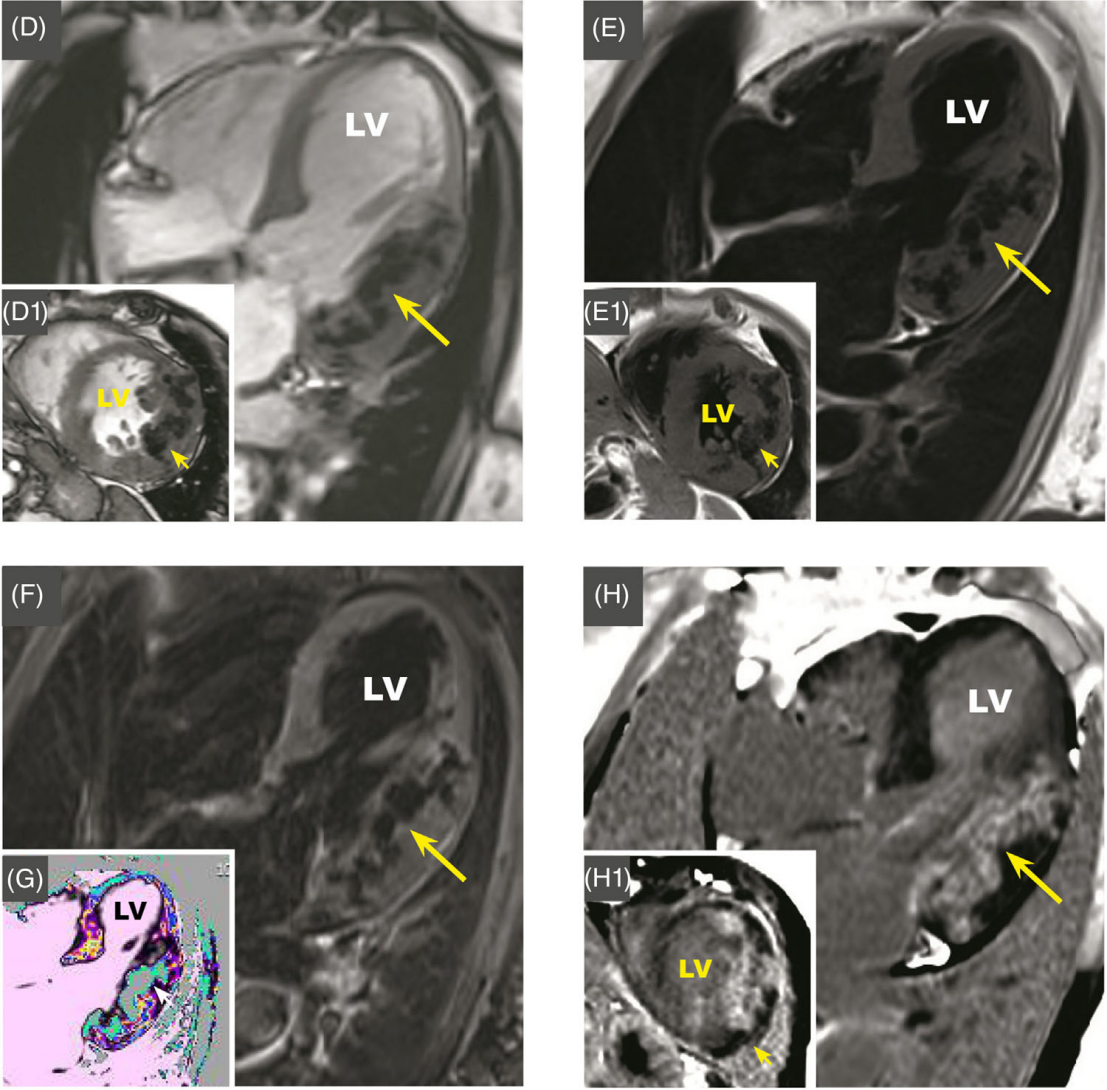
(D) D1: Video 3. CMR SSFP‐cine 4‐chambers and short‐axis views respectively showing lateral and antero‐lateral wall thickening with areas of intra‐myocardial low signal, surrounded by normal myocardium. (E) E1: CMR T1weighted‐spin‐echo 4‐chambers and short‐axis view respectively show thickening of the lateral and antero‐lateral wall with areas of low‐signal. (F) CMR STIR 4‐chambers showing lateral thickening with areas of low‐signal. (G) CMR T1‐native‐mapping demonstrate diffuse septum and lateral fibrosis with low‐signal in correspondence of the calcifications in the lateral wall (gray‐light blue areas). (H) H1: CMR PSIR‐sequences in 4‐chamber view and short‐axis view, respectively. After contrast‐gadolinium injection, intra‐myocardial hypersignal (gadolinium enhancement) is shown in the antero‐lateral wall

An 81‐year‐old woman, affected by Horton disease and with previous history of rheumatic fever, was hospitalized for acute chest pain. Physical examination and EKG were not significant. Chest X‐ray showed a diffuse hypodense lobulated area in the left ventricle (Figure 1A). The echocardiogram revealed diffuse aortic and mitral calcifications with mild stenosis, marked septum, and antero‐lateral asymmetric hypertrophy, with extensive antero‐lateral calcifications (Figure 1B, Video 1). Non‐contrast computed‐tomography (CT) highlighted widespread amorphous confluent calcifications in the left ventricular wall, with septal sparing, extended to the mitral‐aortic annulus and both coronary arteries (Figure 1C‐C1 showing VRT reconstruction and C2 MPR axial‐view, Video 2). Cardiac magnetic resonance (CMR) 3,0 T presented the following: septal hypertrophy, antero‐lateral wall thickening with areas of intra‐myocardial signal‐alteration, surrounded by normal myocardium matching the CT calcifications. Areas of low‐signal were shown in multiple sequences: SSFP‐cine in 4‐chambers (4C) (Figure 2D, Video 3) and in short‐axis (SAX) (Figure 2D1); T1‐weighted‐spin‐echo in 4C (Figure 2E) and SAX (Figure 2E1); STIR in 4C (Figure 2F). T1‐native‐mapping in 4C focused on diffuse septum and lateral fibrosis with low‐signal (550 ms, normal range: 1150–1250 ms) compatible with calcifications (Figure 2G). After contrast‐gadolinium injection, PSIR‐sequence showed intra‐myocardial hypersignal in the lateral wall both in 4C and SAX, Figure 2H‐H1, respectively). Coronary angiography documented significant disease that required revascularization. Extensive intra‐myocardial calcifications are extremely rare.^1^ In our case the diagnostic pathway excluded previous myocardial infarction, myocarditis and calcium‐phosphate disorders. A dystrophic etiology has been supposed. The pathological mechanism for dystrophic calcifications is related to calcium deposition in any dead and dying myocardial tissues. Methastatic calcification are otherwise related to any disturbance in calcium metabolism and can occur with abnormal calcium‐phosphorous homeostasis. There are no standardized imaging features available to classify specific subtypes of intra‐myocardial calcifications.^2^ CT scan is the gold standard examination for the noninvasive detection of myocardial calcifications. CMR with late‐ gadolinium enhancement and native T1/T2 mapping has the unique ability to provide a differential diagnosis in the whole spectrum of myocardial diseases, allowing tissue characterization and hemodynamic assessment. The combination of imaging pattern and potential etiology plays a key role in characterizing calcifications and deriving its clinical impact.

**VIDEO 1:** CMR SSFP‐cine 4‐chambers and short‐axis views respectively showing lateral and antero‐lateral wall thickening with areas of intra‐myocardial low signal, surrounded by normal myocardium

**VIDEO 2:** Non‐contrast CT MPR‐axial view presents widespread calcifications in the left ventricular wall extended to the mitral‐aortic annulus and both coronary arteries

**VIDEO 3:** 2D‐Echocardiogram revealing diffuse aortic and mitral calcifications with mild stenosis, marked septum and antero‐lateral asymmetric hypertrophy, with extensive antero‐lateral calcifications

## CONFLICT OF INTEREST

No conflict of interests exists.
